# Supplementary material for: Exposure of Human Skin Organoids to Low Genotoxic Stress Can Promote Epithelial-to-Mesenchymal Transition in Regenerating Keratinocyte Precursor Cells
Source: Cells. 2020 Aug 18;9(8):1912. doi: 10.3390/cells9081912 (PMC7466070; doi:10.3390/cells9081912)
Supplement: Supplementary file 1 [file cells-09-01912-s001.pdf]

cells

MDPI

Article

Exposure of Human Skin Organoids to Low Genotoxic Stress Can Promote Epithelial-to-Mesenchymal Transition in Regenerating Keratinocyte Precursor Cells

Sophie Cavallero [1,2,3,4,†], Renata Neves Granito [1,2,3,4,‡,§], Daniel Stockholm [5], Peggy Azzolin [1,2,3,4], Michèle T. Martin [1,2,3,4,*] and Nicolas O. Fortunel [1,2,3,4*]

[1] Laboratoire de Génomique et Radiobiologie de la Kératinopoïèse, Institut de Biologie François Jacob, CEA/DRF/IRCM, 91000 Evry, France; socavallero@gmail.com (S.C.); re_neves@yahoo.com.br (R.N.G.); peggy.azzolin@cea.fr (P.A.)
[2] INSERM U967, 92260 Fontenay-aux-Roses, France
[3] Université Paris-Saclay, 75013 Paris 11, France
[4] Université Paris-Diderot, 78140 Paris 7, France
[5] Ecole Pratique des Hautes Etudes, PSL Research University, UMRS 951, Genethon, 91002 Evry, France; stockho@genethon.fr
* Correspondence: michele.martin@cea.fr (M.T.M.); nicolas.fortunel@cea.fr (N.O.F.); Tel: 33-1-60-87-34-91 (M.T.M.); 33-1-60-87-34-92 (N.O.F.); Fax: 33-1-60-87-34-98 (M.T.M. & N.O.F.)
† Present address: DEBR/Unité Rad/Institut de Recherche Biomédicale des Armées, 1 Place du Général Valérie André, 91223 Brétigny sur Orge, France.
‡ Present address: Federal University of São Paulo (UNIFESP), Rua Silva Jardim, 136, Santos 11015020, SP, Brazil.
§ These authors contributed equally.

Received: 18 July 2020; Accepted: 14 August 2020; Published: 18 August 2020

Supplementary Materials

Table of contents.

Figure S1. Absence of epidermis mixing between recipient mice skin and regenerated human epidermis grafts.
Figure S2. Absence of p16 ^{INK4a} in human epidermises regenerated by irradiated and non-irradiated keratinocyte precursors.

Table S1. Absence of p16 ^{INK4a} in human epidermises regenerated by irradiated and non-irradiated keratinocyte precursors.

Supplementary Methods.

References cited in Supplementary Materials.

Supplementary Figures.

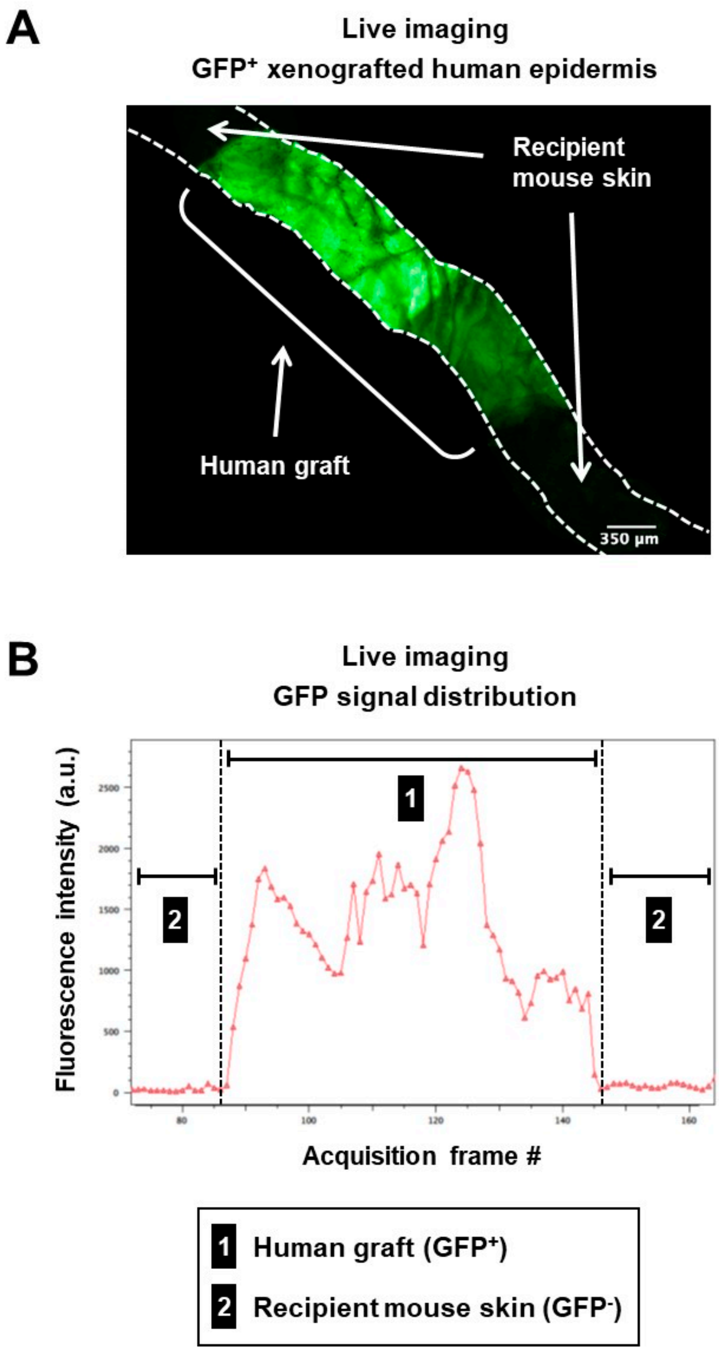

Figure S1. Absence of epidermis mixing between recipient mice skin and regenerated human epidermis grafts. Live imaging performed on grafts generated with [GFP+] transduced keratinocytes previously monitored the coverage of xenograft sites by human regenerated epidermises [1] (see supplementary Methods). (**A**) Representative full-size section of a xenograft site numerically reconstructed by arrangement of successive signal acquisition frames. (**B**). Typical analysis of fluorescence levels (arbitrary units, a.u.) detected within the human graft and within the surrounding mouse skin, indicating a clear-cut border between the skin of recipient mice and the human xenografted skin substitute. No diffuse mixing between the human and mouse epidermises was observed, indicating the absence of recruitment of mouse epithelial cells within human xenografts (n=60 grafts were performed using [GFP+] transduced keratinocytes).

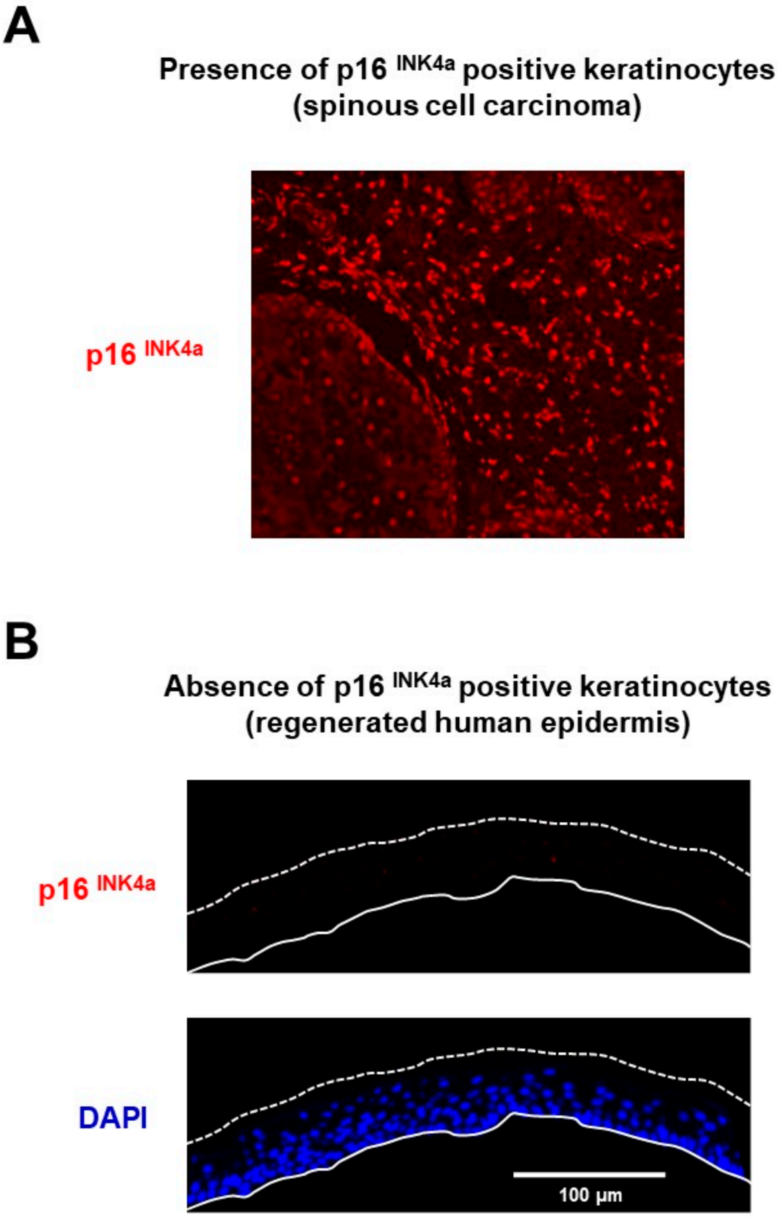

Figure S2. Absence of p16 ^{INK4a} in human epidermises regenerated by irradiated and non-irradiated keratinocyte precursors. The presence or absence of p16 ^{INK4a}, which exerts the function of a stress-induced senescence promoter in keratinocytes [2], was assessed by immunofluorescence using a mouse monoclonal anti-p16 ^{INK4a} [clone 5A8A4] (ThermoFisher scientific, MA5-17093) [see Materials and Methods in the main manuscript]. (**A**) Signal was clearly detected in sections of spinous cell carcinoma, used as a positive control of staining. (**B**) In contrast, p16 ^{INK4a} positive keratinocytes were not visualized in regenerated human epidermis xenografts (time 20 days post-xenografting).

Table S1. Absence of p16 ^{INK4a} in human epidermises regenerated by irradiated and non-irradiated keratinocyte precursors.

**Epidermises regenerated with non-irradiated keratinocyte precursors**			
ROI	Total examined keratinocytes	p16 ^{INK4a} positive keratinocytes	% positive keratinocytes
n°1	124	0	0
n°2	126	0	0
n°3	210	0	0
n°4	122	0	0

Normal areas in epidermises regenerated with irradiated keratinocyte precursors (50 mGy)			
ROI	Total examined keratinocytes	p16 ^{INK4a} positive keratinocytes	% positive keratinocytes
n°1	183	0	0
n°2	135	0	0
n°3	203	0	0
n°4	213	0	0
**Dysplastic areas in regenerated epidermises (50 mGy)**			
ROI	Total examined keratinocytes	p16 ^{INK4a} positive keratinocytes	% positive keratinocytes
n°1	41	0	0
n°2	81	0	0
n°3	32	0	0
n°4	53	0	0

ROI: region of interest (representative areas selected for the analysis).For each context (i.e. non-irradiated control, normal areas from the 50 mGy condition, and dysplastic areas from the 50 mGy condition, analyzed at the time 20 days post-xenografting, four representative epidermis areas (ROI: regions of interest) were selected, in which keratinocytes were identified based on nucleus DAPI staining, and individually examined for p16 ^{INK4a} signal. Keratinocytes exhibiting p16 ^{INK4a} staining were not observed in any of the three tissue contexts, suggesting that senescence is not a major mechanism in dysplasia and EMT development.

Supplementary Methods.

Generation of fluorescent keratinocyte precursors by lentiviral transduction

The lentiviral vector used for the generation of fluorescent [GFP+] transduced keratinocytes was supplied by Vectalys® (Vectalys SA). As described in [1], transduction was performed on cultured holoclone keratinocytes at 50 population doublings after cloning. Transduction was performed at ~20 % confluence. Cells were incubated overnight with lentiviral particles (multiplicity of infection [M.O.I.] at 1) in the presence of hexadimethrine bromide at 8 µg/mL (Sigma-Aldrich). After 3 days, keratinocytes at 80 % confluence were collected and analyzed by flow cytometry (MoFlo, Beckman Coulter). Transduced cells were sorted according to their GFP fluorescence.

Live imaging of xenograft site coverage

Live imaging of xenograft site coverage, based on detection of the GFP fluorescence of transduced human keratinocytes [1], was performed using the fluorescence confocal laser endomicroscopy system Cellvizio® 488 (Mauna Kea Technologies, Paris, France), equipped with the signal acquisition microprobe S-1500. Signal was treated and managed using the IC Viewer software (Mauna Kea Technologies). Full-size sections of xenograft sites were numerically reconstructed by arrangement of successive signal acquisition frames, using the Advanced Mosaicing™ function of the software.

References cited in Supplementary Materials.

1. Fortunel, N.O.; Chadli, L.; Coutier, J.; Lemaître, G.; Auvré, F.; Domingues, S.; Bouissou-Cadio, E.; Vaigot, P.; Cavallero, S.; Deleuze, J-.F.; et al. KLF4 inhibition promotes the expansion of keratinocyte precursors from adult human skin and of embryonic-stem-cell-derived keratinocytes. *Nat. Biomed. Eng*. **2019**, *3*, 985–997.
2. Sasaki, M.; Kajiya, H.; Ozeki, S.; Okabe, K.; Ikebe, T. Reactive oxygen species promotes cellular senescence in normal human epidermal keratinocytes through epigenetic regulation of p16(INK4a.). *Biochem. Biophys. Res. Commun*. **2014**, *452*, 622–628.
